# Alterations in cardiac contractile and regulatory proteins contribute to age‐related cardiac dysfunction in male rats

**DOI:** 10.14814/phy2.70012

**Published:** 2024-08-21

**Authors:** Young Soo Han, Madona Pakkam, Matthew J. Fogarty, Gary C. Sieck, Frank V. Brozovich

**Affiliations:** ^1^ Department of Physiology & Biomedical Engineering Mayo Clinic Rochester Minnesota USA; ^2^ Department of Cardiovascular Diseases Mayo Clinic Rochester Minnesota USA

**Keywords:** Ca^2+^ sensitivity, Ca^2+^ transient, cross‐bridge cycling, myosin binding protein C, myosin heavy chain, titin, troponin

## Abstract

Aging is associated with cardiac contractile abnormalities, but the etiology of these contractile deficits is unclear. We hypothesized that cardiac contractile and regulatory protein expression is altered during aging. To investigate this possibility, left ventricular (LV) lysates were prepared from young (6 months) and old (24 months) Fischer344 rats. There are no age‐related changes in SERCA2 expression or phospholamban phosphorylation. Additionally, neither titin isoform expression nor phosphorylation differed. However, there is a significant increase in β‐isoform of the myosin heavy chain (MyHC) expression and phosphorylation of TnI and MyBP‐C during aging. In permeabilized strips of papillary muscle, force and Ca^2+^ sensitivity are reduced during aging, consistent with the increase in β‐MyHC expression and TnI phosphorylation. However, the increase in MyBP‐C phosphorylation during aging may represent a mechanism to compensate for age‐related contractile deficits. In isolated cardiomyocytes loaded with Fura‐2, the peak of the Ca^2+^ transient is reduced, but the kinetics of the Ca^2+^ transient are not altered. Furthermore, the extent of shortening and the rates of both sarcomere shortening and re‐lengthening are reduced. These results demonstrate that aging is associated with changes in contractile and regulatory protein expression and phosphorylation, which affect the mechanical properties of cardiac muscle.

## 1 INTRODUCTION

During aging, the cardiovascular system undergoes significant remodeling, and diastolic dysfunction represents the primary abnormality of cardiac function (Lakatta, 1990, 2003; North & Sinclair, 2012; Shinmura et al., 2011). Further, the vasculature is also affected by aging, and investigators have documented an increase in vascular stiffness and decrease in NO mediated vasodilatation (Lakatta, 1990, 2003; North & Sinclair, 2012; Shinmura et al., 2011). However, the mechanism(s) that produce age‐related changes in the cardiovascular system is unknown.

Investigators have suggested that diastolic dysfunction that occurs during aging results from a slower rate of reuptake of cytosolic Ca^2+^ by the sarcoplasmic reticulum (Lakatta, 2003; Shinmura et al., 2011), which would slow cardiac relaxation and contribute to the mechanism for diastolic dysfunction. However, a slowing of the rate of decline of the Ca^2+^ transient has not been documented in several studies (Babušíková et al., 2012; Gergs et al., 2019; Jiang et al., 2018). Thus, the contribution of changes in the Ca^2+^ transient to the mechanism producing aging‐related diastolic dysfunction is unclear.

An increase in the expression of the cardiac β‐isoform of the myosin heavy chain (MyHC) has been demonstrated with aging (Carnes et al., 2004; Farrar et al., 1988; Fitzsimons et al., 1999; Lompre et al., 1981), and β‐MyHC has a slower actomyosin ATPase (AMATPase) than α‐MyHC (Alpert et al., 2002; Malmqvist et al., 2004; VanBuren et al., 1995), which would slow cardiac relaxation and contribute to the mechanism that produces diastolic dysfunction. However, the role of other contractile and regulatory proteins in aging associated cardiac dysfunction is unclear. Titin is responsible for majority of the passive stiffness of cardiomyocytes (Granzier & Labeit, 2002; Li et al., 2016; Trombitás et al., 2001) and a shift to the shorter, stiffer N2B isoform increases passive stiffness (Cazorla et al., 2000; Granzier & Labeit, 2002; Linke, 2008; Trombitás et al., 2001), and thus an increase in the expression of N2B titin during aging would result in diastolic dysfunction. Further PKC mediated titin phosphorylation has been demonstrated to increase titin stiffness (Hidalgo et al., 2009; LeWinter & Granzier, 2013), and an increase in titin phosphorylation at S11878 and/or S12022 would produce diastolic dysfunction and could also contribute to the mechanism responsible for diastolic dysfunction during aging. Additionally, changes in phosphorylation of both TnI and MyBP‐C are well known to alter cardiac contractile properties (Solaro et al., 2010); TnI phosphorylation has been demonstrated to decrease Ca^2+^ sensitivity of force, or shift the force versus Ca^2+^ relationship to the right (Kentish et al., 2001; Layland et al., 2005), while MyBP‐C phosphorylation has been demonstrated to both increase the number of myosin S1 heads interacting with actin and the rate of the AMATPase (Kensler et al., 2017; Moss et al., 2015). Thus, changes in the phosphorylation or isoform expression of titin as well as the phosphorylation of the regulatory proteins TnI and MyBP‐C could participate in the mechanism producing age‐related cardiac dysfunction.

The present study was designed to test the hypothesis that the expression of cardiac contractile and regulatory proteins is altered during aging, which contributes to the mechanisms underlying age‐related cardiac contractile abnormalities.

## 2 MATERIALS AND METHODS

### 2.1 Animals

The experimental protocol was approved by the Mayo Clinic Institutional Animal Care and Use Committee and conformed to the guidelines of the National Institutes of Health. Male Fischer344 rats were studied at three different ages (6, 18, and 24 months). Prior to the experiments, rats were anesthetized using an intramuscular injection of ketamine (60 mg/kg) and xylazine (2.5 mg/kg), then the heart was rapidly excised, and the animals were euthanized by exsanguination.

### 2.2 Immunoblotting

As previously described (Degen et al., 2015; Given et al., 2007; Konik et al., 2013; Yuen et al., 2009), immunoblotting was used to determine protein expression. Briefly, left ventricular cardiac muscle samples were homogenized in SDS sample buffer, and total protein (TP) extract was resolved by SDS‐PAGE, with sample loading normalized to TP within the band calculated from stain‐free precast gels (Biorad Cat#64551870) as previously described (Han et al., 2021; Schaible et al., 2016; Yap et al., 2020). After SDS‐PAGE, proteins were transferred onto an immunoblot membrane (Biorad Cat#1629177) and anti‐myosin binding protein C (703,574, Invitrogen), −phospholamban (ab2199626, Abcam), ‐S16 phospho‐phospholamban (ab15000, Abcam), ‐SERCA2 (ab137020, Abcam), ‐TnI (10‐T79C, Fitzgerald), ‐S23/24 phosphoTnI (4004, Cell Signaling), ‐MyBP‐C (ab133499, Abcam) and phosphorylation specific‐phosphoMyBP‐C (‐phosphoS273, ‐phosphoS282 and ‐phosphoS302) antibodies (Sadayappan et al., 2009) were used to visualize the proteins. The resulting immunoblots were scanned and analyzed using ImageLab software and protein expression was normalized for TP as previously described (Han et al., 2021; Schaible et al., 2016; Yap et al., 2020). Cardiac MyHC expression was determined as previously described (Ogut et al., 2007) using the method initially reported by Talmadge and Roy (Talmadge & Roy, 1993). In brief, 8% gels (50:1; acrylamide/bis) with 30% glycerol were loaded with samples and run at 6 mA for 22 h at room temperature, and subsequently immunoblotted with anti‐cardiac MyHC (ab50967, Abcam) and anti‐cardiac β‐MyHC (AMB1548, Millipore) antibodies (Han et al., 2021). Both total cardiac MyHC expression and cardiac β‐MyHC were normalized for TP, as described (Han et al., 2021; Schaible et al., 2016; Yap et al., 2020); the ratio of cardiac β‐MyHC to total cardiac MyHC is (cardiac β‐MyHC/TP)/(total cardiac MyHC/TP), which is given as β‐MyHC/MyHC. MyBP‐C was visualized with SYPRO Ruby staining and phosphorylation of MyBP‐C was examined using Pro‐Q Diamond phosphoprotein staining as previously described (Han et al., 2021). Titin isoform expression was determined using a previously published protocol (Tatsumi & Hattori, 1995; Warren et al., 2003). Briefly, frozen tissue samples were homogenized in 50 mM Tris‐SDS buffer (pH 6.8) containing 8 μg/mL leupeptin (Peptin Institute, Japan) and a phosphatase inhibitor cocktail consisting of 10 μL/mL PIC I (P2850, Sigma) and 10 μL/mL PIC II (P5726, Sigma), heated, centrifuged, and separated on agarose‐strengthened 2% SDS‐PAGE gels. Samples were loaded in duplicate and run at 5 mA for 16 hours. Gels were washed and stained with SYPRO Ruby. The optical volume of protein bands (integrated optical density) was determined using ImageQuant TL software. Titin N2BA titin isoform expression was normalized to total titin expression (N2B + N2BA). Titin phosphorylation was determined with the same protocol, but gels were first stained with Pro‐Q Diamond phosphoprotein stain for 1 hour, and then washed and subsequently stained with SYPRO Ruby. Titin phosphorylation was expressed relative to the SYPRO Ruby‐stained TP signal to correct for differences in sample loading.

### 2.3 Papillary muscle mechanics

Thin strips (~400μm × 200μm, 1.5 mm in length) of the anterior papillary muscle were prepared and permeabilized as previously described (Han et al., 2013; Han & Ogut, 2011). The preparations were mounted using aluminum foil T‐clips (Ogut & Brozovich, 2000, 2003a, 2003b; Rhee & Brozovich, 2003) in a muscle mechanics workstation between a length driver (600A; Aurora Scientific, Canada) and force transducer (AE802; SensorOne, Sausilito, CA). Temperature was maintained at 15 ± 0.1°C. The preparation was activated by changing [Ca^2+^] and the relationship between [Ca^2+^] and force was fit using the Hill equation (Brozovich et al., 1988). Additionally, at maximal Ca^2+^ activation, rate constant of force redevelopment after quick release and restretch (k_tr_) was determined using previously published protocols (Ogut & Brozovich, 2000, 2003a); briefly the increase in force was fit to a double exponential, and the initial rate of the rapid increase in force was taken as k_tr_.

### 2.4 Ca^2+^ transients and shortening in cardiomyocytes

The protocol for isolation of cardiomyocytes has been previously described in detail (Schaible et al., 2016). Briefly, using a modified Langendorff perfusion system, the heart was cannulated via the aorta and perfused with pre‐warmed (37°C) and oxygenated Tyrode's solution for 5 min and then Type II collagenase solution for 10 min. The enzymatically digested heart was minced in Type II collagenase solution for 5 min and filtered through a stainless‐steel mesh (25μmx25μm), and the filtrate was centrifuged at 1000 rpm for 1 min and incubated with Kraft‐Brühe solution at 37°C for 30 min. Then, the cardiomyocytes were incubated in Cardiac Myocyte Medium (CAT#6201, ScienCell) at 37°C for another 30 min. Ca^2+^ and sarcomere length (SL) were measured using an IonOptix system (IonOptix LCC, Milton, MA) as previously described (Schaible et al., 2016). Briefly, isolated cardiomyocytes were loaded with 1 μM Fura‐2 am for 10 min at 37°C. After loading, the cardiomyocytes were placed in a tissue chamber and perfused with Ca^2+^ Tyrode's solution at 37°C and aerated with a 95% O_2_−5% CO_2_ for at least 10 min. Isolated cardiomyocytes were stimulated with 250 mA current, 5 ms duration pulses at a frequency of 0.5 Hz using the MyoPacer (IonOptix), and Ca and SL were simultaneously recorded using IonWizard software. Fura‐2 fluorescence was excited using alternating 340 and 380 nm wavelengths and emission fluorescence was collected at 510 nm using a photomultiplier tube. The [Ca^2+^] was determined based on calibration of the ratio of Fura‐2 fluorescence (*R* = 340 nm/380 nm) using the equation described by Grynkiewicz et al. (1985; Williams & Fay, 1990).

### 2.5 Quantification of hypertrophy and fibrosis

The extent of cardiac hypertrophy and fibrosis were determined from hematoxylin–eosin (HE) and Masson's trichome stained sections of cardiac left ventricular muscle as previously described (Lin et al., 2020). Cardiac muscle was embedded in paraffin and then thin sections (10 μm) were stained with HE or Masson's trichrome (Han et al., 2021). Photomicrographs were obtained, and for each slide, at least 6 fields were analyzed. Quantification of myocyte cross‐sectional area and interstitial fibrosis was performed using ImageJ analysis software (Version 1.49, NIH, Bethesda, MD).

### 2.6 Statistical analysis

All data in the text are presented as mean ± SEM (*n* = number of animals) and data in the figures are displayed using Box plots. For the mechanical experiments, a single papillary muscle was used per animal. Based on a power analysis (power = 80%, α = 0.05) of primary outcome measures, the experiments included 6 animals per age group. Differences between age groups (6, 18, and 24 months) were compared using a one‐way ANOVA, and if significant differences were found, Student's *t*‐test was used post hoc to compare values with a *p* < 0.05 level significance.

## 3 RESULTS

### 3.1 Age‐related changes in cardiac contractile and regulatory protein expression

**FIGURE 1:**
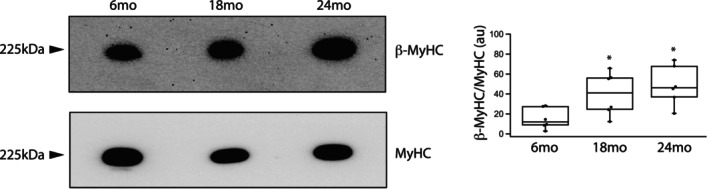
Myosin expression increases during aging. Immunoblotting demonstrates that the expression of β‐MyHC increases during aging. Box plot shows that compared to 6 months, the relative expression of β‐MyHC (β‐MyHC/MyHC) increases at both 18 and 24 months; *n* = 6 for each age group (*p < 0.05 vs. 6 months).

**FIGURE 2:**
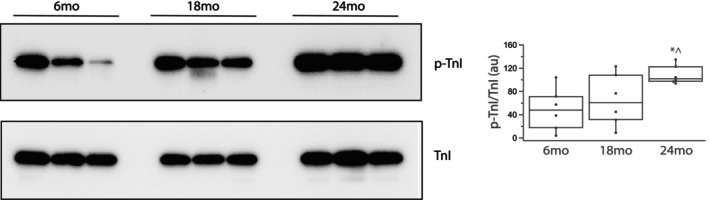
TnI phosphorylation increases during aging. Immunoblots show expression of both TnI and S23/24 phosphorylated TnI (p‐TnI) for 6, 18, and 24 months. Box plot summarizes the data and demonstrates that TnI phosphorylation is higher at 24 months than at either 6 and 18 months; *n* = 6 per group (*, *p* < 0.05 vs. 6 months; ^*p* < 0.05 vs. 18 months).

**FIGURE 3:**
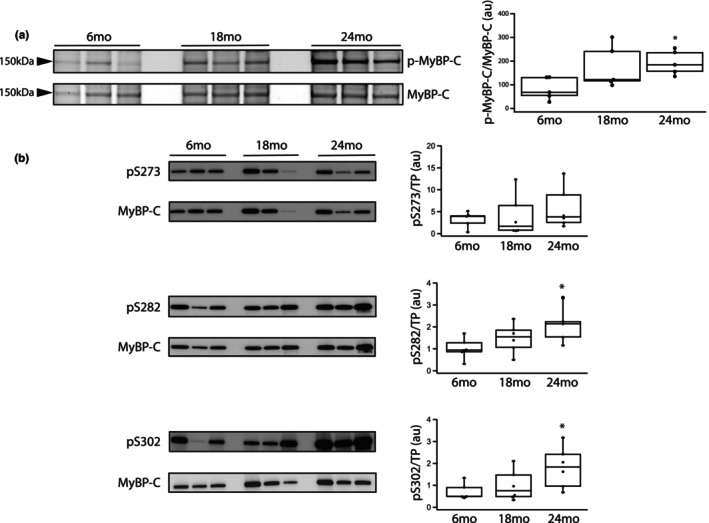
MyBP‐C phosphorylation increases during aging. (a) SYPRO‐Ruby staining (bottom) and Pro‐Q Diamond staining (upper) were used to quantify MyBP‐C and phosphorylated MyBP‐C (p‐MyBP‐C) expression. Box plot summarizes the data and demonstrates that phosphorylation of MyBP‐C (p‐MyBP‐C/MyBP‐C) is significantly higher at 24 months compared to 6 months; *n* = 6 at each age, *, *p* < 0.05. (b) Immunoblots demonstrate MyBP‐C expression as well as S273, S282 and S302 MyBP‐C phosphorylation at 6, 18, and 24 months. Box plots show protein expression normalized for total protein (TP), as previously described (Schaible et al., 2016; Yap et al., 2020), and summarize the data. Both S282 and S302 MyBP‐C phosphorylation, normalized for total protein (TP), are significantly higher at 24 months compared to 6 months (*n* = 6 at each age); *p < 0.05 vs. 6 months.

Myosin heavy chain expression as well as the phosphorylation of both TnI and MyBP‐C were examined in both young and old rats (*n* = 6 per group). Total MyHC was normalized to TP (Figure S1) as previously described (Han et al., 2021; Schaible et al., 2016; Yap et al., 2020) and is similar at all ages (*p* > 0.05); MyHC/TP expression is 3.5 ± 0.8au (6 months), 2.2 ± 0.5au (18 months), 3.4 ± 0.9au (24 months). However, the expression of cardiac β‐MyHC/TP increases with age; 0.61 ± 0.31au at 6 months, 0.82 ± 0.28au at 18 months and 1.40 ± 0.31au at 24 months (*p* = 0.038). Thus, the ratio of β‐MyHC/MyHC increases with age (Figure 1); 15 ± 4au at 6 months, 40 ± 8au at 18 months (*p* = 0.0015) and 49 ± 8au at 24 months (*p* = 0.018). We also found that S23/24 phosphorylation of TnI (p‐TnI) increases during aging (Figure 2); p‐TnI/TnI is 49 ± 15au at 6 months, 66 ± 18au at 18 months and 108 ± 7au at 24 months (Figure 2); although TnI phosphorylation is similar at 6 months and 18 months, it is significantly higher at 24 months when compared to both 6 months (*p* = 0.0004) and 18 months (*p* = 0.0017). We defined total MyBP‐C phosphorylation with ProQ Diamond staining and immunoblotting to assess both total MyBP‐C expression and MyBP‐C phosphorylation at S273, S282, and S302 (Sadayappan et al., 2009). Our data demonstrate that normalized MyBP‐C expression (MyBP‐C/TP) is similar at 6 months (1.7 ± 0.1au) and 18 months (2.2 ± 0.3) but is significantly higher at 24 months when compared to 6 months (2.7 ± 0.2au, *p* = 0.0001, Figure 3). Total MyBP‐C phosphorylation increased during aging (Figure 3a); (p‐MyBP‐C/MyBP‐C) is significantly higher at 24 months (190 ± 20au) compared to 6 months (110 ± 30au, *p* = 0.030). We also defined MyBP‐C phosphorylation at PKA phosphorylation sites (Hou et al., 2022; Mamidi et al., 2016; McNamara et al., 2019; Tong et al., 2008) during aging (Figure 3b). Although S273MyBP‐C/TP phosphorylation is similar in all age groups (0.75 ± 0.03au vs 0.91 ± 0.10au vs 0.74 ± 0.07au, *p* > 0.05), MyBP‐C phosphorylation at both S282 and S302 increases with aging (6 months vs. 24 months, *p* < 0.05); S282MyBP‐C phosphorylation (normalized for TP) is 1.0 ± 0.2au (6 months), 1.5 ± 0.3au (18 months) and 1.9 ± 0.4au (24 months, *p* = 0.031) and S302MyBP‐C phosphorylation (normalized for TP) is 0.73 ± 0.17au (6 months), 0.98 ± 0.31au (18 months) and 1.8 ± 0.4au (24 months, *p* = 0.019).

**FIGURE 4:**
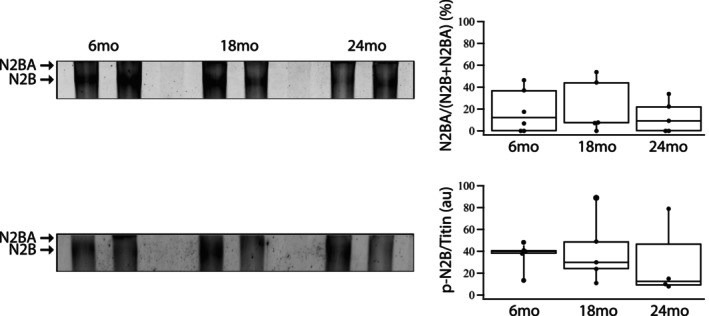
Titin isoform expression and phosphorylation during aging. Upper panel shows SYPRO Ruby stained gel shows titin isoform expression; N2BA (upper band) and N2B (lower band) at 6, 18, and 24 months (*n* = 6 per group). Lower panel shows ProQ Diamond stained gel demonstrates titin phosphorylation. Box plots summarizes the data; there is no significant difference (*p* > 0.05) in relative titin isoform expression (N2BA/[N2B + N2BA]) or titin phosphorylation (p‐Titin/Titin, ProQ Diamond/SYPRO Ruby) with aging.

We also determined titin isoform expression, which has been demonstrated to regulate the majority of the stiffness of cardiomyocytes (Granzier & Labeit, 2002; Li et al., 2016; Trombitás et al., 2001). Titin expression is predominantly N2B, and titin isoform expression did not change during aging (Figure 4). Additionally, we determined titin phosphorylation using ProQ Diamond staining. Relative titin phosphorylation, N2B phosphorylation/total titin expression (ProQ Diamond/SYPRO Ruby) did not change with aging.

In addition, we examined the expression and phosphorylation of proteins that are known to regulate the Ca^2+^ transient. Neither the expression of SERCA2 (Figure S2) nor phosphorylation of PLB (Figure S3) changes during aging.

### 3.2 Age‐related changes in cardiac muscle mechanical properties

**FIGURE 5:**
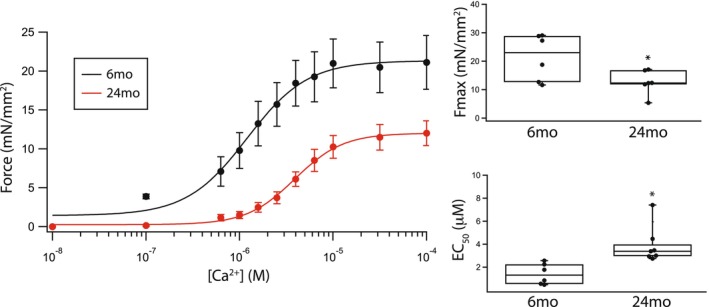
Ca^2+^ sensitivity and maximum force of cardiac muscle decrease during aging. Data of force vs. [Ca^2+^] were fit to the Hill Equation to determine Ca^2+^ sensitivity (EC_50_). At 24 months (red, *n* = 7), compared to 6 months (black, *n* = 6), both steady state maximal force and Ca^2+^ sensitivity are significantly lower. Box plot summarizes the data (*p < 0.05).

**FIGURE 6:**
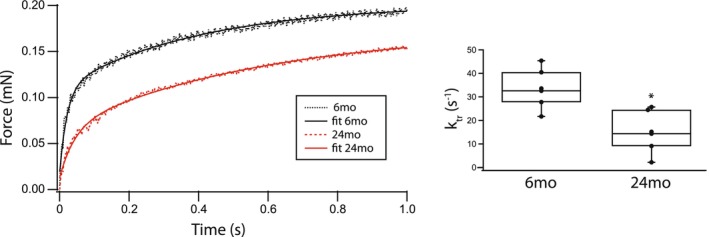
k_tr_ decreases during aging. The rate of cross‐bridge cycling was investigated at maximal Ca^2+^ activation by determining k_tr_ in papillary muscle strips. At 24 months (red, *n* = 6), compared to 6 months (black, *n* = 6), k_tr_ is significantly slower. Box plot summarizes the data (*p < 0.05).

The mechanical properties of cardiac muscle reflected by the maximum Ca^2+^ activated force, the Ca^2+^ sensitivity of force and k_tr_ of permeabilized papillary muscle strips are altered during aging. Both steady state maximal Ca^2+^ activated force (21.1 ± 3.3mN/mm^2^ vs. 11.9 ± 1.0mN/mm^2^, *p* = 0.007, Figure 5) and Ca^2+^ sensitivity (EC_50_: 1.41 ± 0.40 μM vs. 3.92 ± 0.67 μM, *p* = 0.003, Figure 5) are lower in skinned papillary muscle strips prepared from the elderly animals (24 months, *n* = 7) compared to 6 months rats (*n* = 6). The overall rate of cross‐bridge cycling the rate of crossbridge attachment and detachment; f + g (Huxley, 1974), was investigated with measurements of k_tr_ (Brenner & Eisenberg, 1986). Our data demonstrates that k_tr_ is slower in papillary muscle of 24 months animals (15 ± 5 s^−1^, *n* = 6), compared to young rats (33 ± 5 s^−1^, *n* = 6, *p* = 0.009, Figure 6).

### 3.3 Age‐related changes in Ca^2+^ transients and SL shortening in cardiomyocytes

**FIGURE 7:**
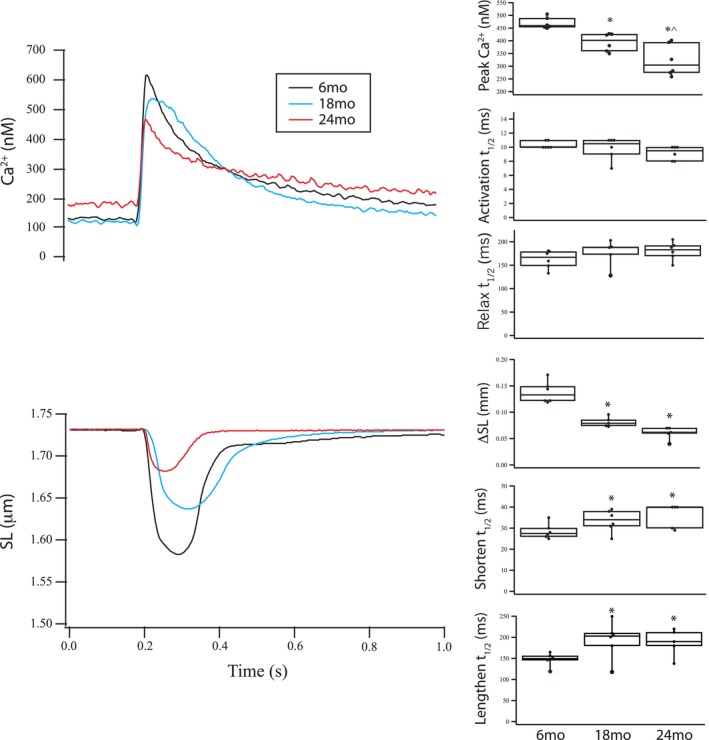
The Ca^2+^ transient and SL shortening of single cardiomyocytes are altered during aging. Representative Ca^2+^ transient and SL shortening of single cardiomyocytes in response to electrical field stimulation, 6 months, black; 18 months, blue; 24 months, red. Box plots (mean ± SEM, *p < 0.05 vs. 6 months, ^*p* < 0.05 v18mo) demonstrate data from 10 isolated myocytes per animal (*n* = 6) from each age group (6, 18, and 24 months). For the Ca^2+^ transient, peak Ca^2+^ declined with aging. However, there was no difference in the kinetics of the Ca^2+^ transient. The extent of SL shortening decreased with aging, and similarly, both the rate of shortening (Activation *t*_1/2_) as well as the rate of relaxation (Relax *t*_1/2_) decrease with aging.

The impact of aging on the relationship between Ca^2+^ transient and SL shortening responses was examined in isolated cardiomyocytes (Figure 7). With aging, the peak Ca^2+^ is reduced from 470 ± 10 nM at 6 months to 390 ± 15 nM at 18 months and to 320 ± 30 nM at 24 months (6 vs. 24 months, *p* = 0.0001, 18 vs. 24 months, *p* = 0.017; *n* = 6 per age group). However, there is no difference in the kinetics of the Ca^2+^ transient; the half time of the time to peak Ca^2+^ (*t*_1/2_; 10 ± 0.2 ms vs. 10 ± 0.7 ms vs. 9 ± 0.4 ms) and the half time of decay (*t*_1/2_; 162 ± 8 ms vs. 180 ± 10 ms vs. 180 ± 8 ms) were similar in all age groups (Figure 7).

In contrast, all parameters of SL shortening are altered during aging (Figure 7). There is a significant decrease in the extent of SL shortening (0.14 ± 0.01 μm vs. 0.07 ± 0.01 μm vs. 0.07 ± 0.01 μm; 6 vs. 18 months, *p* = 0.0005, 6 vs. 24 months, *p* = 0.0006) as well as the rate of both shortening (*t*_1/2_; 28 ± 2 ms vs. 34 ± 2 ms vs. 36 ± 2 ms; 6 vs. 18 months, *p* = 0.042, 6 vs. 24 months, *p* = 0.015) and re‐lengthening (*t*_1/2_; 150 ± 7 ms vs. 190 ± 20 ms vs. 190 ± 10 ms; 6 vs. 18 months, *p* = 0.017, 6 vs. 24 months, *p* = 0.012).

### 3.4 Cardiac hypertrophy and fibrosis

**FIGURE 8:**
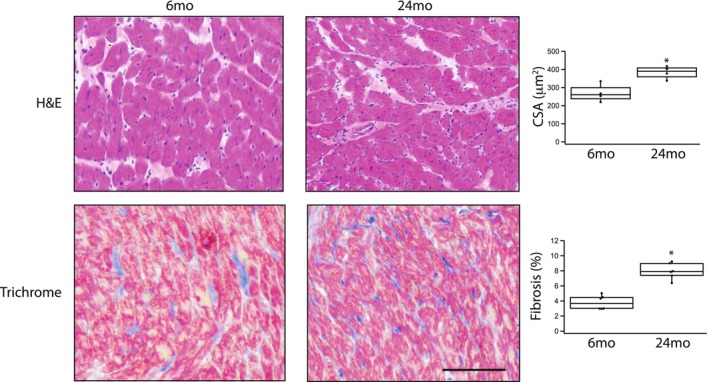
Myocyte cross‐sectional area and cardiac fibrosis increase with age. Representative tissue sections of cardiac muscle stained with hematoxylin–eosin (H&E) or Masson's trichome (Trichrome); scale bar, 100 μm (for all images). Box plots summarize the data for both myocyte cross‐section area (CSA) and cardiac tissue fibrosis. Both myocyte cross‐section and cardiac muscle fibrosis are higher at 24 months compared to 6 months (*p < 0.05).

We also defined the cross‐sectional area of the myocytes as well as cardiac muscle fibrosis from HE and Masson's trichrome stained sections of cardiac muscle (Figure 8). Comparing 6 months (*n* = 6) and 24 months (*n* = 6), aging is associated with an increase in both myocyte cross‐sectional area (270 ± 25 μm^2^ vs. 380 ± 17 μm^2^, *p* = 0.004) and cardiac muscle fibrosis (3.8 ± 0.3% vs. 8.0 ± 0.4%, *p* = 0.00001).

## 4 DISCUSSION

The present study demonstrates that the expression of both cardiac contractile and regulatory proteins is altered during aging, and these changes contribute to the contractile deficits that are associated with aging. Our data show that during aging there is a significant increase in the expression of cardiac β‐MyHC (Figure 1) and phosphorylation of both TnI (Figure 2) and MyBP‐C (Figure 3). Similar to our results, others have demonstrated an increase in cardiac β‐MyHC during aging (Carnes et al., 2004; Farrar et al., 1988; Fitzsimons et al., 1999; Lompre et al., 1981). Cardiac α‐MyHC has been demonstrated to have a ~ 2–3 fold higher actin‐activated ATPase and velocity of actin movement in the motility assay than cardiac β‐MyHC (Alpert et al., 2002; Malmqvist et al., 2004; VanBuren et al., 1995). Additionally, in large mammals, cardiac α‐MyHC produces an ~2x higher force compared to cardiac β‐MyHC, while there is no difference in force for α‐MyHC and β‐MyHC in smaller mammals including the mouse (Malmqvist et al., 2004) and rat (Fitzsimons et al., 1998; Sugiura et al., 1995). Thus, the increase in the expression of β‐MyHC in cardiac muscle during aging documented in the present study would be expected to result in a reduction in k_tr_ (Figure 6) and a decrease the rates of SL shortening and relaxation of single cardiomyocytes (Figure 7). Aging is also well known to be associated with an increase in myocyte size and cardiac fibrosis (Lakatta, 2003), which is consistent with our results (Figure 8). Further, despite the increase in myocyte size, total MyHC expression did not change, and thus, the number of myosin filaments per myocyte cross‐section would decrease and coupled with the increase in cardiac fibrosis would be expected to contribute to the decrease in the maximally Ca^2+^ activated force/cross‐section in 24 months rats (Figure 5).

In mice, TnI phosphorylation has been reported to decrease (Salcan et al., 2020) and also not change (Kane et al., 2020) during aging. The differences in these results may be due to differences in the techniques used to define TnI phosphorylation; data comparing the ratio of the density of the band at ~24 kDa on a ProQ Diamond versus Coomassie stained gel (Kane et al., 2020) are not as specific as immunoblotting (Salcan et al., 2020). Our data demonstrates S23/24 TnI phosphorylation increases during aging in the rat (Figure 2). Cardiac β‐MyHC isoform is not expressed in either young or elderly mice (Malmqvist et al., 2004; Ng et al., 2018), which may explain the difference in our results in rats vs others in mice. Other investigators did not demonstrate a decrease in either force or Ca^2+^ sensitivity during aging, but aging was associated with a decrease in *V*_max_ and the rate of force development (Fitzsimons et al., 1999), which is consistent with our results. However, TnI phosphorylation was not measured in this study (Fitzsimons et al., 1999). Nonetheless, an increase in cardiac TnI phosphorylation (S23/24) has been demonstrated to both decrease Ca^2+^ sensitivity or shift the force vs Ca^2+^ relationship to the right (Kentish et al., 2001; Layland et al., 2005) and reduce the rate of cardiac muscle relaxation (Kentish et al., 2001). These data are consistent with our current observations demonstrating increased TnI phosphorylation with aging (Figure 2), as well as a decreased Ca^2+^ sensitivity of force (Figure 5) observed in 24 months rats and decreased rate of SL shortening and re‐lengthening in cardiomyocytes isolated from 24 months rats (Figure 7). Similar to our results in single cardiomyocytes (Figure 7), others have demonstrated that the duration of cardiac muscle contraction is prolonged in elderly rodents (Lakatta et al., 1975) and the increase in the expression of β‐MyHC (Figure 1) and phosphorylation of TnI (Figure 2) documented with aging in the present study would be expected to contribute to the slower rates of shortening and re‐lengthening observed in both cardiomyocytes (Figure 7) and trabeculae (Lakatta et al., 1975).

Our data also demonstrate that MyBP‐C phosphorylation increases during aging (Figure 3a). The preponderance of data suggest that TnI phosphorylation is the primary regulator of Ca^2+^ sensitivity, while MyBP‐C phosphorylation is more important for the regulation of the kinetics of the AMATPase (Solaro et al., 2010). MyBP‐C is known to both activate the thin filament and stabilize the super‐relaxed state (SRX) of myosin (Harris, 2020). Dephosphorylated MyBP‐C has been demonstrated to stabilize the SRX state and PKA mediated phosphorylation of MyBP‐C allows myosin S1 heads to move away from the thick filament backbone and interact with actin (Colson et al., 2008; Kensler et al., 2017; McNamara et al., 2019). Thus, PKA mediated phosphorylation of MyBP‐C would be expected to increase the number of myosin heads interacting with actin and accelerate the AMATPase (Kensler et al., 2017; Moss et al., 2015). Consistent with this hypothesis are results demonstrating that phosphorylation of MyBP‐C increases the speed of actin movement in the motility assay (Previs et al., 2012) and force redevelopment in permeabilized cardiac muscle (Stelzer et al., 2006). In transgenic mice expressing MyBP‐C with S‐A mutations of PKA phosphorylation sites, activation of PKA did not increase the rates of stretch activation (Tong et al., 2008) or force relaxation (Tanner et al., 2021; Tong et al., 2008). Additionally in the mice expressing S‐A mutant MyBP‐C, PKA did not increase the kinetics of cardiac muscle contraction (Tong et al., 2015). Further, there was an increase in the rate of force relaxation as well as enhanced diastolic function in transgenic mice expressing MyBP‐C with S‐D mutations compared to S‐A mutant MyBP‐C (Rosas et al., 2015). Ponnam et al. (2019) have demonstrated that PKA phosphorylates MyBP‐C in sequential order S282 followed by S273 and then S302, which agrees with data demonstrating MyBP‐C phosphorylation at S282 increases phosphorylation at S273 (Gupta et al., 2013). Further, data suggests that S273 phosphorylation may be primary for regulating the interaction of MyBP‐C with myosin while S282 phosphorylation is most important for regulating MyBP‐C's ability to activate the thin filament (Ponnam et al., 2019). However, other data suggest that S302 is the critical phosphorylation site for mediating PKA dependent increases in contractile kinetics and force in cardiac muscle MyBP‐C enhancement of cardiac function (Mamidi et al., 2017). In contrast, in mice expressing mutant MyBP‐C with either nonphosphorylatable (A) or phosphomimicking (D) mutations at S273, S282 and S302, data suggest that S282 phosphorylation is the primary regulator of MyBP‐C effects on inotropy (Gupta et al., 2013). Our data demonstrate that MyBP‐C phosphorylation increases at both S282 and S302 with aging (Figure 3b), which would be expected to increase force, as well as the rate of cross‐bridge cycling rate and cardiac relaxation. However, during aging, our data show decreases in maximal force (Figure 5), k_tr_ (Figure 6), and the rate of both SL shortening and re‐lengthening (Figure 7). In mice, MyBP‐C phosphorylation has been reported to increase during aging (Kane et al., 2020), but in contrast to these results, another group has demonstrated that MyBP‐C phosphorylation decreases with aging, and transgenic mice expressing S‐D mutant MyBP‐C had less aging associated deterioration of systolic and diastolic function (Rosas et al., 2019). However, unlike other mammals, including the rat (Figure 1), cardiac β‐MyHC isoform is not expressed in either young or elderly mice (Malmqvist et al., 2004; Ng et al., 2018), which may explain the difference in these results. Thus during aging, our results in rats demonstrating increased phosphorylation of cardiac MyBP‐C at S282 and S302 (Figure 3b) could represent a compensatory mechanism, counteracting the decreases in force and cross‐bridge cycling rates that occur in response to the increases cardiac fibrosis, β‐MyHC expression and phosphorylation of TnI.

Titin is responsible for majority of the passive stiffness of cardiomyocytes (Granzier & Labeit, 2002; Li et al., 2016; Trombitás et al., 2001) and a shift to the shorter, stiffer N2B isoform increases passive stiffness (Cazorla et al., 2000; Granzier & Labeit, 2002; Linke, 2008; Trombitás et al., 2001), which would decrease the rate of myocyte shortening as well as relaxation. We found that titin expression is predominantly N2B at all ages (Figure 4). Cazorla et al (2000) reported that N2BA expression varies widely among species with 5% N2BA in the mouse, 0% N2BA in the rat, 50% in human and 90% in bovine cardiac tissue. However, relative N2BA expression is not a consistent when comparing different studies. Salcan et al (2020) reported that in the mouse, N2BA expression was 20% of total titin expression and in healthy humans, N2BA represented ~40% of total titin expression. Similarly, others have demonstrated that N2BA expression in human ventricular tissue can range from 40% (Kötter et al., 2013) to 60% of total titin expression (Hamdani et al., 2013; Zakeri et al., 2016). Titin phosphorylation has also been demonstrated to alter its stiffness; PKA and PKG mediated phosphorylation decrease titin stiffness (Krüger et al., 2009; Krüger & Linke, 2009; Linke, 2008; Yamasaki et al., 2002) while PKC mediated phosphorylation increases titin‐based stiffness (Hidalgo et al., 2009). Our data demonstrate that titin isoform expression did not change (Figure 4), which is similar to that reported by others in mice (Salcan et al., 2020), and suggest changes titin isoform expression do not contribute to the mechanism producing age‐related cardiac dysfunction. These investigators (Salcan et al., 2020) also demonstrated that passive tension of the cardiac myofilaments isolated from mice did not change during aging and although they did not report total titin phosphorylation, they showed that during aging, there were site specific changes in titin phosphorylation; phosphorylation was lower at S4010 (PKA site), phosphorylation was higher at S4099 (PKG site) while phosphorylation at S11878 and S12022 (PKC sites) was similar. Our data demonstrate that neither titin isoform expression nor titin phosphorylation changes during aging (Figure 4). These data suggest that there may be site‐specific changes in titin phosphorylation as reported by Salcan et al (2020). However, since the passive stiffness in cardiomyocytes does not change with aging (Salcan et al., 2020), any changes in phosphorylation at the PKA and PKG sites that decrease stiffness (Krüger et al., 2009; Krüger & Linke, 2009; Linke, 2008; Yamasaki et al., 2002) must be balanced and/or offset by changes in phosphorylation at the PKC sites which increase titin stiffness (Hidalgo et al., 2009). These results would predict that total titin phosphorylation would be constant, which agrees with our results (Figure 4).

SERCA2 and PLB are important for the regulation of the Ca^2+^ transient (MacLennan & Kranias, 2003), and aging has been demonstrated to be associated with a decrease in activity of the Ca^2+^‐ATPase (Lakatta, 2003). In cardiac tissue lysates from young or elderly rats, our data demonstrated that there is no difference in SERCA2 expression (Figure S2) or PLB phosphorylation (Figure S3). These results are similar to those reported by others in both rodents (Babušíková et al., 2012; Jiang et al., 2018) and in human (Gergs et al., 2019) cardiac muscle. However, there are conflicting reports that demonstrate aging is associated with a decrease in SERCA2 expression (Froehlich et al., 1978; Knyushko et al., 2005; Shinmura et al., 2011) and PLB expression and phosphorylation (Shinmura et al., 2011). Although others have also reported that SERCA2 and PLB expression does not change during aging, oxidative stress has been suggested to reduce Ca^2+^‐ATPase activity (Babušíková et al., 2012; Knyushko et al., 2005; Xu et al., 2006). Our data from single cardiomyocytes data show that the Ca^2+^ transient had a lower peak, but there was no difference in the kinetics of the Ca^2+^ transient (Figure 7). Further, aging was associated with a decreased extent of SL shortening, as well as the rate of both shortening and re‐lengthening (Figure 7), which is similar to the results of others (Kane et al., 2020; Shinmura et al., 2011). Kane et al (Kane et al., 2020) reported that the Ca^2+^ transient is altered during aging with decreases in peak Ca^2+^ and the rates of rise and fall of the Ca^2+^ transient. The results reported by Shinmura et al (2011) are similar to ours; aging was not associated with a change in the magnitude or the rate of the rise of the Ca^2+^ transient, but the rate of the decline of the Ca^2+^ transient was significantly slower in elderly animals.

In summary, our results demonstrate that aging is associated with changes in contractile protein expression and phosphorylation, which affect the mechanical properties of cardiac muscle. Our data do not support that a change in the kinetics of the Ca^2+^ transient underlies the contractile abnormalities associated with aging. However, the lower peak Ca^2+^ would be expected to reduce the extent of SL shortening and peak twitch force. Our results also demonstrate that aging is associated with changes in the expression and phosphorylation of both cardiac contractile and regulatory proteins: the increase in the expression of β‐MyHC as well as the phosphorylation of TnI would result in a decrease in Ca^2+^ sensitivity, cross‐bridge cycling rate and rates of SL shortening and re‐lengthening, contributing to the mechanism for the contractile abnormalities that occur during aging. However, the increase in phosphorylation of MyBP‐C could represent a mechanism to compensate for age‐related deterioration of cardiac function.

## AUTHOR CONTRIBUTIONS

Young Soo Han designed and performed the experiments, analyzed the data and wrote the manuscript; Madona Pakkam participated in performing and analyzing the immunoblots; Matthew J. Fogarty performed and analyzed the histology; Gary C. Sieck participated in designing the study and revising the manuscript; Frank V. Brozovich designed and supervised the experiments and revised the manuscript.

## FUNDING INFORMATION

This study was supported by a Mayo Clinic CV Prospective Research Award (FVB).

## CONFLICT OF INTEREST STATEMENT

The authors have no conflict of interest to declare.

## ETHICS STATEMENT

The experimental protocol was approved by the Mayo Clinic Institutional Animal Care and Use Committee and conformed to the guidelines of the National Institutes of Health.

## Supporting information


**Figure S1:** Total Protein. Criterion TGX stain‐free gel demonstrating total protein for loading control and normalization.

**Figure S2:** SERCA2 expression. Immunoblot demonstrates SERCA2 expression at 6, 18, and 24 months. Bar graph summarizes the data; there was no significant difference in SERCA2 expression (SERCA2/TP) at 6 months (black, *n* = 6), 18 months (blue, *n* = 6) or 24 months (red, *n* = 6).

**Figure S3:** PLB expression and phosphorylation. Western blot of PLB (bottom panel) and phosphorylated PLB (p‐PLB, upper panel) at 6, 18, and 24 months. Bar graph summarizes the data; there was no significant difference in PLB phosphorylation (p‐PLB/PLB) at 6 months (black, *n* = 6), 18 months (blue, *n* = 6) or 24 months (red, *n* = 6).

**Figure S4:** Uncut images. Uncut immunoblots for MyHC (β‐MyHC, MyHC and lane 4) purified skeletal muscle (Sk) MyHC, TnI (TnI and phosphor‐TnI), phosphorylated MyBPC (p273, p2982 and p302) and titin SYPRO Ruby stained gel.

## Data Availability

Data are available on request.
