# Supplementary figures and images for: Alterations in cardiac contractile and regulatory proteins contribute to age‐related cardiac dysfunction in male rats
Source: Physiol Rep. 2024 Aug 21;12(16):e70012. doi: 10.14814/phy2.70012 (PMC11338742; doi:10.14814/phy2.70012)

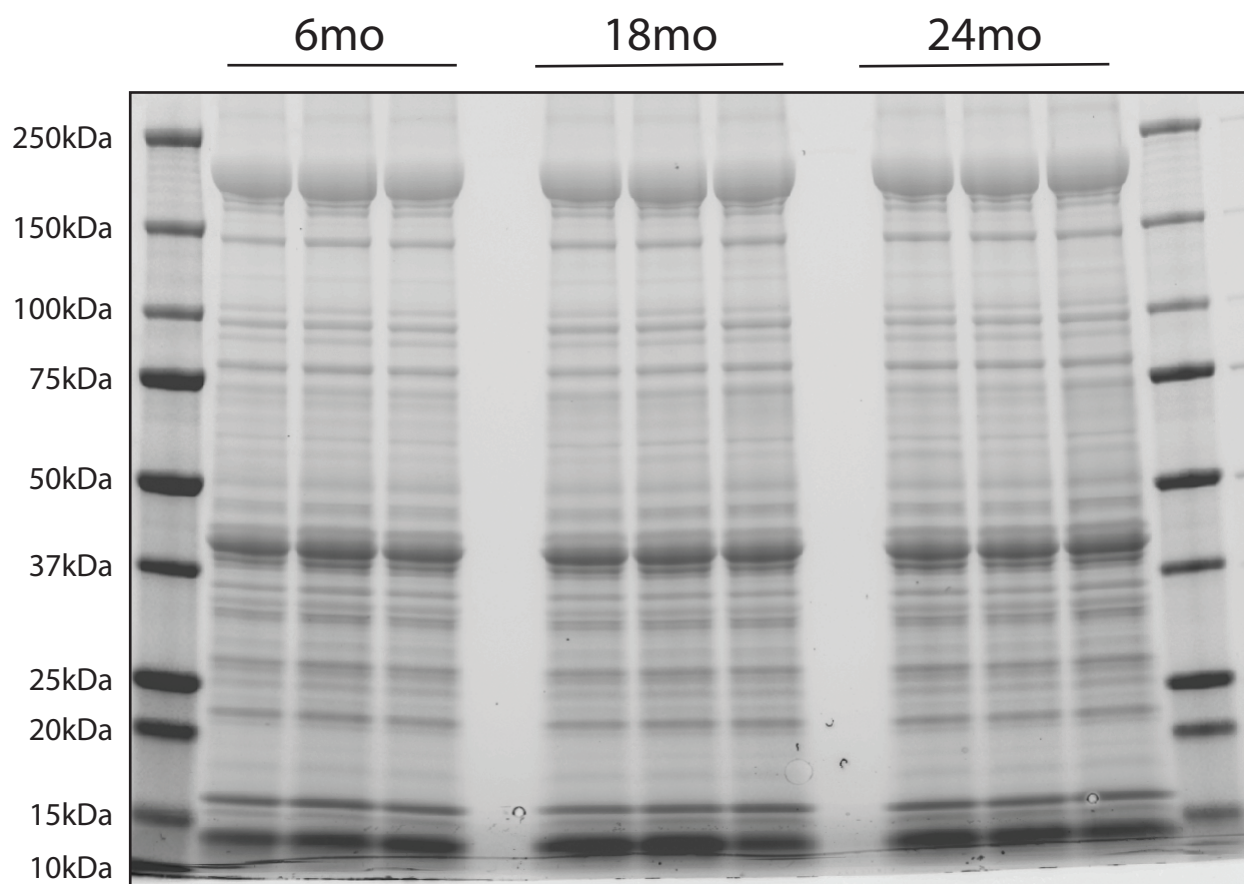

Supplement: Supplementary file 1 — Figure S1: Total Protein. Criterion TGX stain‐free gel demonstrating total protein for loading control and normalization. [file PHY2-12-e70012-s004.pdf]

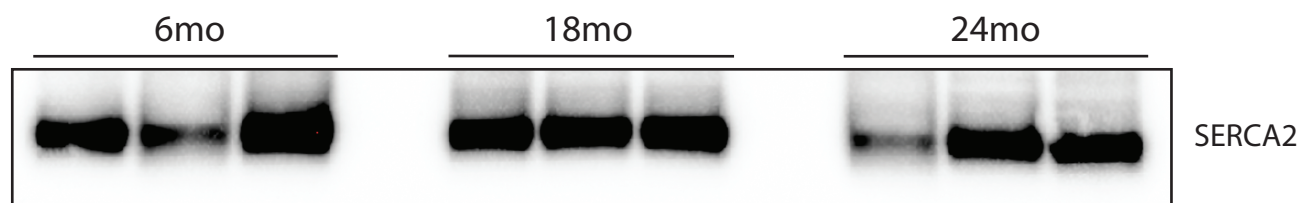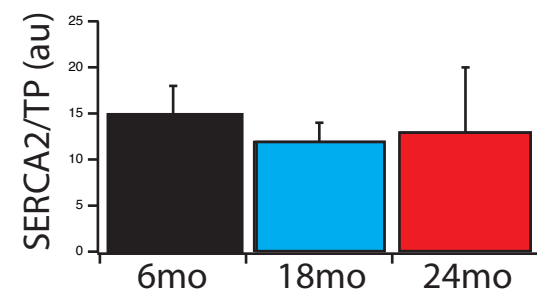

Supplement: Supplementary file 2 — Figure S2: SERCA2 expression. Immunoblot demonstrates SERCA2 expression at 6, 18, and 24 months. Bar graph summarizes the data; there was no significant difference in SERCA2 expression (SERCA2/TP) at 6 months (black, n = 6), 18 months (blue, n = 6) or 24 months (red, n = 6). [file PHY2-12-e70012-s001.pdf]

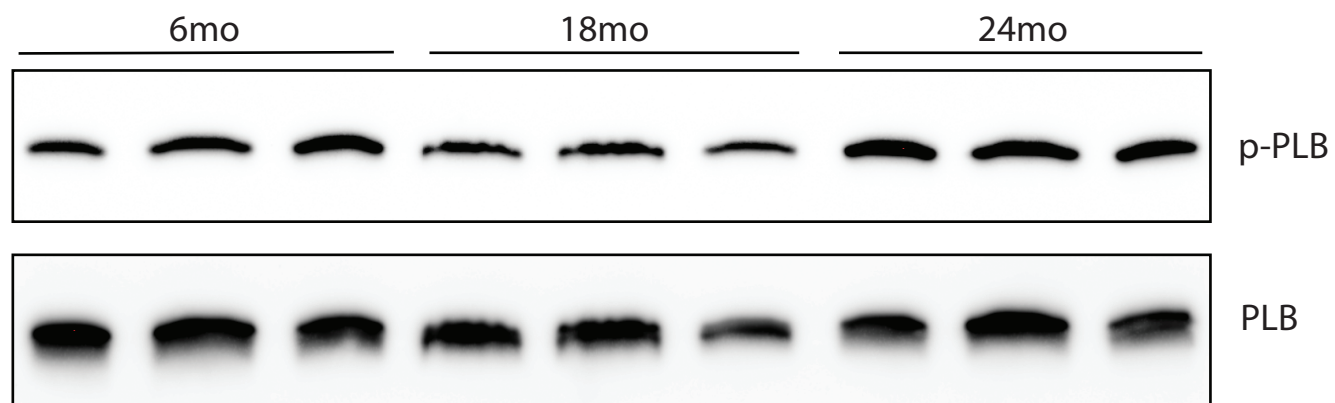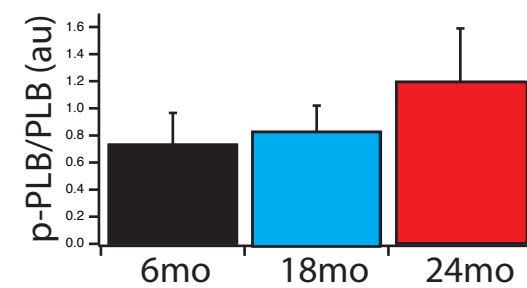

Supplement: Supplementary file 3 — Figure S3: PLB expression and phosphorylation. Western blot of PLB (bottom panel) and phosphorylated PLB (p‐PLB, upper panel) at 6, 18, and 24 months. Bar graph summarizes the data; there was no significant difference in PLB phosphorylation (p‐PLB/PLB) at 6 months (black, n = 6), 18 months (blue, n = 6) or 24 months (red, n = 6). [file PHY2-12-e70012-s002.pdf]

MyHC

6mo 12mo 24mo 5k MyHC

$\beta$ -MyHC

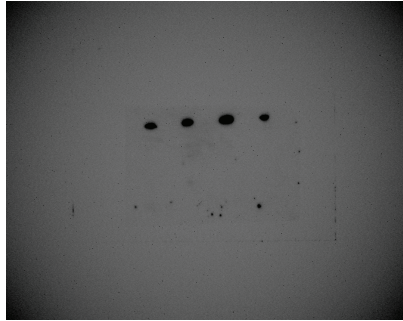

TnI

P-TnI

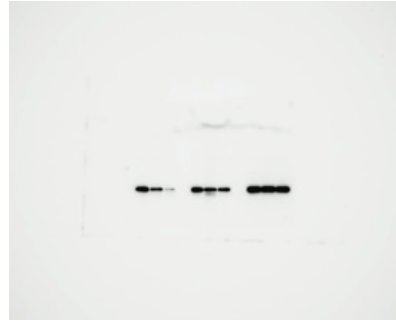

MyBPC

pS273

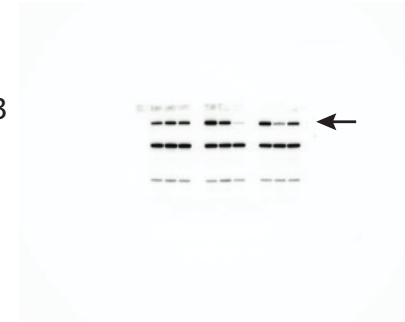

MyHC

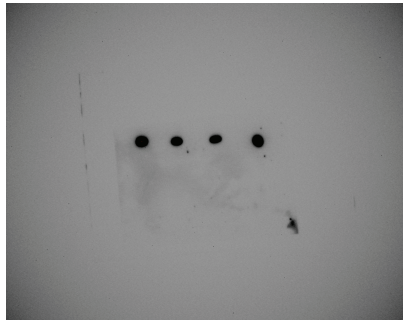

TnI

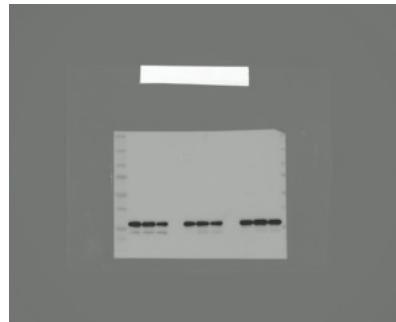

pS282

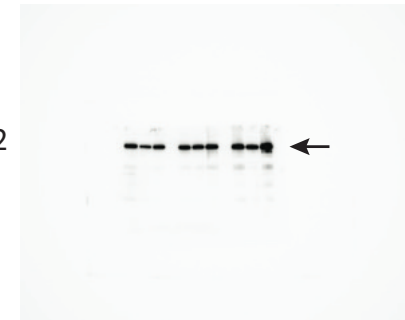

Titin

N2BA  
N2B

MyHC

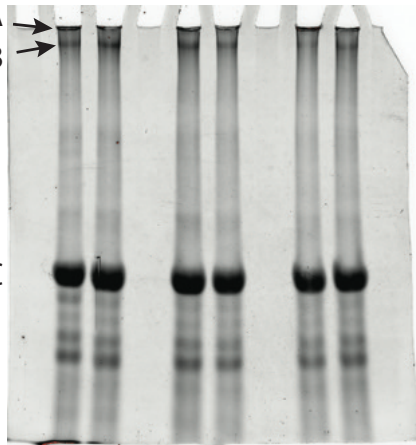

pS302

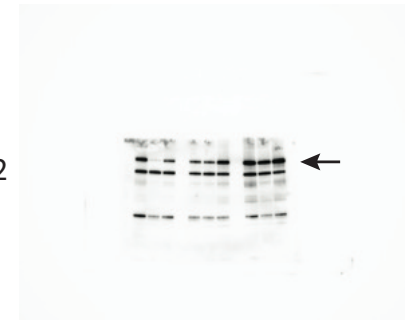

Supplement: Supplementary file 4 — Figure S4: Uncut images. Uncut immunoblots for MyHC (β‐MyHC, MyHC and lane 4) purified skeletal muscle (Sk) MyHC, TnI (TnI and phosphor‐TnI), phosphorylated MyBPC (p273, p2982 and p302) and titin SYPRO Ruby stained gel. [file PHY2-12-e70012-s003.pdf]
